# Limits of long-term selection against Neandertal introgression

**DOI:** 10.1073/pnas.1814338116

**Published:** 2019-01-15

**Authors:** Martin Petr, Svante Pääbo, Janet Kelso, Benjamin Vernot

**Affiliations:** ^a^Department of Evolutionary Genetics, Max Planck Institute for Evolutionary Anthropology, 04103 Leipzig, Germany

**Keywords:** Neandertal, selection, introgression, modern human, demography

## Abstract

Several studies have suggested that introgressed Neandertal DNA was subjected to negative selection in modern humans. A striking observation in support of this is an apparent monotonic decline in Neandertal ancestry observed in modern humans in Europe over the past 45,000 years. Here, we show that this decline is an artifact likely caused by gene flow between modern human populations, which is not taken into account by statistics previously used to estimate Neandertal ancestry. When we apply a statistic that avoids assumptions about modern human demography by taking advantage of two high-coverage Neandertal genomes, we find no evidence for a change in Neandertal ancestry in Europe over the past 45,000 years. We use whole-genome simulations of selection and introgression to investigate a wide range of model parameters and find that negative selection is not expected to cause a significant long-term decline in genome-wide Neandertal ancestry. Nevertheless, these models recapitulate previously observed signals of selection against Neandertal alleles, in particular the depletion of Neandertal ancestry in conserved genomic regions. Surprisingly, we find that this depletion is strongest in regulatory and conserved noncoding regions and in the most conserved portion of protein-coding sequences.

## Significance

Since the discovery that all non-Africans inherit 2% of their genomes from Neandertal ancestors, there has been a great interest in understanding the fate and effects of introgressed Neandertal DNA in modern humans. A number of recent studies have claimed that there has been continuous selection against introgressed Neandertal DNA over the last 55,000 years. Here, we show that there has been no long-term genome-wide removal of Neandertal DNA, and that the previous result was due to incorrect assumptions about gene flow between African and non-African populations. Nevertheless, selection did occur following introgression, and its effect was strongest in regulatory regions, suggesting that Neandertals may have differed from humans more in their regulatory than in their protein-coding sequences.

Interbreeding between Neandertals and modern humans ∼55,000 y ago has resulted in all present-day non-Africans inheriting at least 1–2% of their genomes from Neandertal ancestors (1, 2). There is significant heterogeneity in the distribution of this Neandertal DNA across the genomes of present-day people (3, 4), including a reduction in Neandertal alleles in conserved genomic regions (3). This has been interpreted as evidence that some Neandertal alleles were deleterious for modern humans and were subject to negative selection following introgression (3, 5). Several studies have suggested that low effective population sizes (*N*_e_) in Neandertals led to decreased efficacy of purifying selection and the accumulation of weakly deleterious variants. Following introgression, these deleterious alleles, along with linked neutral Neandertal alleles, would have been subjected to more efficient purifying selection in the larger modern human population (6, 7).

**Fig. 1.**
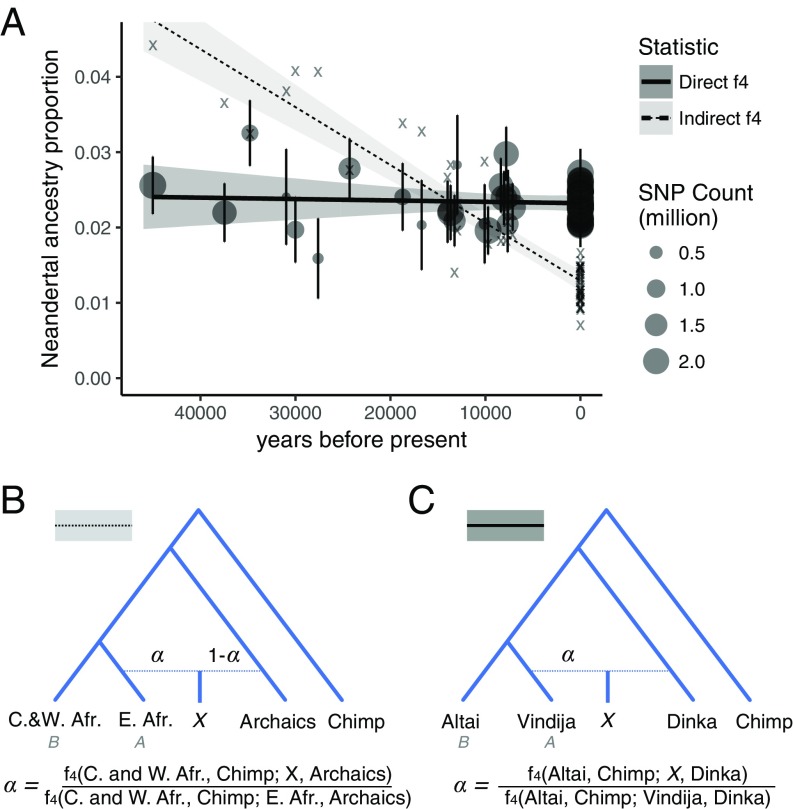
Direct and indirect *f*_4_-ratio estimates of Neandertal ancestry. (*A*) Best linear fits for indirect and direct *f*_4_-ratio estimates of Neandertal ancestry in ancient and modern West Eurasians (solid points for direct *f*_4_-ratio, “x” for indirect *f*_4_-ratio). Shaded areas are 95% CIs (*SI Appendix*, section S1). (*B*) Tree model and formula used for the indirect *f*_4_-ratio. (*C*) Tree model and formula used for the direct *f*_4_-ratio. Present-day individuals are West Eurasians from the SGDP panel, excluding individuals from the Near East (Neandertal ancestry for all West Eurasians shown in *SI Appendix*, Fig. S7).

In apparent agreement with this hypothesis, a study of Neandertal ancestry in a set of anatomically modern humans from Upper-Paleolithic Europe used two independent statistics to conclude that the amount of Neandertal DNA in modern human genomes decreased monotonically over the last 45,000 y (Fig. 1*A*, dashed line) (8). This decline was interpreted as direct evidence for continuous negative selection against Neandertal alleles in modern humans (8–11). However, it was not formally shown that selection on deleterious introgressed variants could produce a decline in Neandertal ancestry of the observed magnitude. Nevertheless, this decrease in Neandertal ancestry—together with the suggestion of a higher burden of deleterious alleles in Neandertals—are now commonly invoked to explain the fate of Neandertal ancestry in modern humans (9–12).

Here, we reexamine estimates of Neandertal ancestry in ancient and present-day modern humans, taking advantage of a second high-coverage Neandertal genome that recently became available (13). This allows us to avoid some key assumptions about modern human demography that were made in previous studies. Our analysis shows that the Neandertal ancestry proportion in Europeans has not decreased significantly over the last 45,000 y. Using simulations of selection and introgression, we show that a model of weak selection against deleterious Neandertal variation also does not predict significant changes in Neandertal ancestry during the time period covered by existing ancient modern human samples. In contrast, these simulations do predict a depletion of Neandertal ancestry around functional genomic regions. We then use our updated Neandertal ancestry estimates to examine the genomic distribution of introgressed Neandertal DNA and find that selection against introgression was strongest in regulatory and conserved noncoding regions compared with protein-coding sequence (CDS), suggesting that regulatory differences between Neandertals and modern humans may have been more extreme than protein-coding differences.

## Results

### Previous Neandertal Ancestry Estimate.

A number of methods have been developed to quantify Neandertal ancestry in modern human genomes (14). Among the most widely used is the *f*_4_-ratio statistic, which measures the fraction of drift shared with one of two parental lineages to determine the proportion of ancestry, α, contributed by that lineage (Fig. 1 and *SI Appendix*, Fig. S1) (15, 16). Although they have been used to draw inferences about gene flow between archaic and modern human populations, *f*_4_-ratio statistics are known to be sensitive to violations of the underlying population model (15). Estimating α, the proportion of ancestry in *X* contributed by a lineage *A*, requires a sister lineage *B* to lineage *A* which does not share drift with *X* after separation of *B* from *A* (*SI Appendix*, Fig. S1). Fu et al. (8) used an *f*_4_-ratio statistic to infer the contribution from an archaic lineage by first estimating the proportion of East African ancestry in a non-African individual *X*, under the assumption that Central and West Africans (*B*) are an outgroup to the East African lineage (*A*) and to the modern human ancestry in non-Africans. Defining this East African ancestry proportion as α = *f*_4_(C. and W. Africans, Chimp; *X*, Archaics)*/f*_4_(C. and W. Africans, Chimp; E. Africans, Archaics), the proportion of archaic ancestry was then calculated simply as 1 − α, under the assumption that all ancestry that is not of East African origin must come from an archaic lineage (8). We refer to this statistic as an “indirect *f*_4_-ratio.”

Given the sensitivity of the *f*_4_-ratio method to violations of the underlying population models (15), we explored the validity of assumptions on which this calculation was based. In addition to the topology of the demographic tree, which has recently been shown to be incorrect (17), the indirect *f*_4_-ratio assumes that the relationship between Africans and West Eurasians has remained constant over time (8). However, our understanding of modern human history and demography have been challenged by new fossil discoveries (18) and the analysis of ancient DNA, with several studies documenting previously unknown migration events in both West Eurasia (19) and Africa (17, 20, 21). Furthermore, an *f*_4_ statistic sensitive to changes in the relationships between West Eurasians and various African populations [formulated as *f*_4_(Ust’-Ishim, *X*; African, Chimp), where *X* is a West Eurasian individual] shows increasing allele sharing between West Eurasians and Africans over time (*SI Appendix*, Fig. S2*A*). In contrast, *f*_4_(Ust’-Ishim, Papuan; African, Chimp) is not significantly different from zero (*|Z| <* 1 when using Dinka, Yoruba, or Mbuti in the third position of the *f*_4_ statistic), demonstrating that this trend is not shared by all non-Africans.

**Fig. 2.**
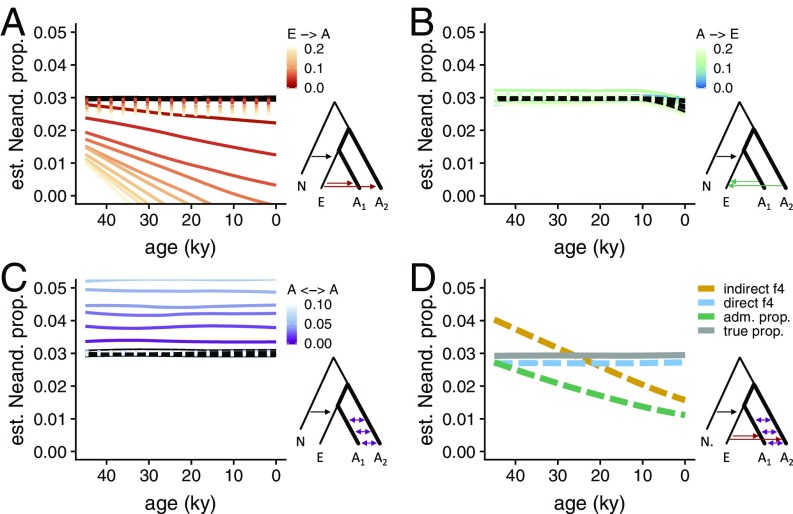
Neandertal ancestry estimates in neutral simulations of migration. Genomic data were simulated under a base model of 3% Neandertal admixture, *N*_e_ = 6,000 in Europeans and *N*_e_ = 14,000 in two African populations (*SI Appendix*, Fig. S8, section S2). (*A*–*C*) The effect of three migration parameters on direct and indirect *f*_4_-ratio estimates of Neandertal ancestry (dotted and solid colored lines, respectively). “Total migration” is shown, that is, *gm*, where *g* is generations of migration, and *m* is the proportion of the target population composed of migrants in each generation. If present, continuous migration between A_1_ and A_2_ begins 40 kya and migration between Europe and Africa begins 5 kya. True Neandertal ancestry proportions are shown in black, and closely match the direct *f*_4_*-*ratio estimates (mean absolute difference from truth for indirect *f*_4_-ratio is 2.6%, 0.12%, and 2.8% for *A*, *B*, and *C* respectively; for direct *f*_4_-ratio 0.25%, 0.05%, and 0.06%). (*D*) Simulations of an example demographic model with migration parameters 0.09, 0.0, and 0.1 for E → A, A → E, and A ↔ A, respectively, which approximate the empirical direct and indirect *f*_4_-ratios (Fig. 1*A*).

To evaluate the sensitivity of the indirect *f*_4_-ratio to migration events, we performed neutral simulations of Neandertal, West Eurasian, and African demographic histories (Fig. 2). All simulations included introgression from Neandertals into West Eurasians, and varying levels of migration between Africans and West Eurasians, and between African populations. We find that gene flow from West Eurasians into Africans leads to misestimates of Neandertal ancestry when using the indirect *f*_4_-ratio statistic, and results in the incorrect inference of a continuous decline in Neandertal ancestry. This decline is not observed in the true simulated Neandertal ancestry (Fig. 2*A*). The magnitude of this bias depends on the total amount of West Eurasian gene flow into Africa, with larger amounts leading to apparent steeper declines (Fig. 2*A*). Additionally, gene flow between the two African populations used in the indirect *f*_4_*-*ratio calculation leads to overestimation of the true level of Neandertal ancestry (Fig. 2*C*). Overall, we find that a combination of West Eurasian migration to Africa and gene flow between African populations can produce patterns that are very similar to those observed in the empirical data (Fig. 2*D* and *SI Appendix*, Fig. S3*A*). However, we caution that effective population sizes and the timing of migration also affect these estimates (*SI Appendix*, Fig. S3), and that there are likely many additional models that match the empirical data.

We note that an independent statistic, using a different set of genomic sites in the same ancient individuals, had been used as a second line of evidence for an ongoing decrease in Neandertal ancestry (8). This statistic, which we refer to as the “admixture array statistic,” measures the proportion of Neandertal-like alleles in a given sample at sites where present-day Yoruba individuals carry a nearly fixed allele that differs from homozygous sites in the Altai Neandertal (22). Much like the indirect *f*_4_-ratio, we find that the admixture array statistic is affected by gene flow from non-Africans into Africans and incorrectly infers a decline in the Neandertal ancestry over time (Fig. 2*D*).

Given the indirect *f*_4_-ratio’s sensitivity to modern human demography, combined with our incomplete understanding of human migrations, we sought to reevaluate the patterns of Neandertal ancestry in modern humans in a more robust manner.

### A Robust Statistic to Estimate Neandertal Ancestry.

The recent availability of a second high-coverage Neandertal genome allows us to estimate Neandertal ancestry using two Neandertals—an individual from the Altai Mountains, the so-called “Altai Neandertal” (23) and an individual from the Vindija Cave in Croatia, the so-called “Vindija Neandertal” (13). Specifically, we can estimate the proportion of ancestry coming from the Vindija lineage into a modern human (*X*) using the Altai Neandertal as a second Neandertal in an *f*_4_-ratio calculated as *f*_4_(Altai, Chimp; *X*, African)*/f*_4_(Altai, Chimp; Vindija, African), which we refer to as a “direct *f*_4_-ratio” (Fig. 1*C* and *SI Appendix*, Fig. S1). Note that unlike the indirect *f*_4_-ratio described previously, the *f*_4_-ratio in this formulation does not make assumptions about deep relationships between modern human populations (Fig. 1*C* and *SI Appendix*, Fig. S1). Instead, it assumes that any Neandertal population that contributed ancestry to *X* formed a clade with the Vindija Neandertal. Recent analyses showed that this is the case for all non-African populations studied to date, including the ancient modern humans in this study (13, 24). When calculated on the simulations described above, we find that the direct *f*_4_-ratio is more robust than the indirect *f*_4_-ratio (Fig. 2). In fact, its temporal trajectory always closely matches the true simulated Neandertal ancestry trajectory, regardless of the specific parameters of gene flow between non-Africans and Africans (Fig. 2). We note that gene flow from West Eurasians into Africans, which introduces introgressed Neandertal alleles into Africa, produces a slight underestimate of Neandertal ancestry in all samples (Fig. 2*A*). This is in agreement with empirical direct *f*_4_-ratio estimates, which vary depending on the African population used in the calculation, with African populations known to carry West Eurasian ancestry (e.g., Mozabite, Saharawi) (17, 25) generating the lowest estimates (*SI Appendix*, Fig. S4). Crucially, when we use the direct *f*_4_-ratio to estimate the trajectory of Neandertal ancestry in ancient and present-day Europeans, we observe nearly constant levels of Neandertal ancestry over time (Fig. 1*A*, points and solid line) and find that a null model of zero slope can no longer be rejected (Fig. 1*A*, *P* = 0.36, estimated via resampling as described in *SI Appendix*, section S1).

We note that these estimates are based on a relatively small number of individuals, especially for older time points, and that the CIs are wide. For example, we cannot reject a linear decline in Neandertal ancestry of approximately half a percent over the timespan of this dataset (95% CI −0.51–0.37%). Additionally, these analyses are performed on SNPs that were ascertained largely in present day individuals. To examine the effects of such ascertainment, we split the dataset based on the ascertainments used and recalculated the direct and indirect *f*_4_-ratios on each of the subsets (*SI Appendix*, Fig. S5). Although the slopes show some variability, in all but one ascertainment subset the direct *f*_4_-ratio cannot reject a slope of 0, whereas the indirect *f*_4_-ratio consistently rejects a slope of 0, suggesting that these results are robust to the effects of ascertainment (*SI Appendix*, Fig. S5). In addition to calculating direct *f*_4_-ratio estimates, we estimated Neandertal ancestry proportions using the qpAdm method (26) and obtained similar results (null model of zero slope using Neandertal ancestry point estimates cannot be rejected with *P* = 0.17).

Our observation that there has been no change in Neandertal ancestry over the past 45,000 y has several implications for our understanding of the fate of Neandertal DNA in modern humans. First, it constrains the timescale during which selection could have significantly affected the average genome-wide Neandertal ancestry in modern humans, an issue addressed below in more detail. Second, a previous analysis of a 40 ky old individual (“Tianyuan”) from East Asia applied the indirect *f*_4_-ratio statistic to estimate his Neandertal ancestry proportion at 5% (27). When we apply the direct *f*_4_-ratio statistic for this individual, we arrive at a value of ∼2.1% (using Dinka as the African group in the calculation). Third, it has consequences for the so-called “dilution” hypothesis, which suggests that lower levels of Neandertal ancestry in Europeans compared with East Asians can be explained by dilution of Neandertal ancestry in Europeans due to admixture with a hypothetical Basal Eurasian population that carried little to no Neandertal ancestry (19, 28). Previous studies have found Basal Eurasian ancestry in all modern and some ancient Europeans [in this study, four ancient individuals show evidence of Basal Eurasian ancestry: Satsurblia (15 kya), Kotias (10 kya), Ranchot88 (10 kya), and Stuttgart (8 kya), *SI Appendix*, Fig. S6] (8, 19). Our finding that there is no ongoing decline in Neandertal ancestry in Europeans suggests that Neandertal ancestry in Europe has not been diluted in a significant way by gene flow from Basal Eurasians. Specifically, we find no difference in Neandertal ancestry in European individuals with and without Basal Eurasian ancestry (direct *f*_4_-ratio mean 2.31% vs. 2.38%, respectively; *P* = 0.36). However, given the small number of relevant samples we also cannot exclude that there could be up to 13% less Neandertal ancestry in individuals with Basal Eurasian ancestry, or as much as 6% more Neandertal ancestry in individuals without Basal Eurasian ancestry (95% CI).

In contrast, we do find that present-day Near Easterners carry significantly less Neandertal ancestry than Europeans (direct *f*_4_-ratio mean 2.03% vs. 2.33%; *P* = 0.001; *SI Appendix*, Fig. S7*A*). Furthermore, present-day populations in the Near East show even stronger signals of admixture with a deeply divergent modern human lineage than observed in the rest of West Eurasians (*SI Appendix*, Fig. S7*B*), suggesting that they carry additional ancestry components that are not present in Europe and that could potentially contribute to lower Neandertal ancestry in the Near East. We note, however, that a simple model of admixture from Africa into Near East would be expected to produce a similar *f*_4_ statistics difference between Near East and the rest of West Eurasia and could also explain lower values of Neandertal ancestry in this population.

### Long-Term Dynamics of Selection Against Introgressed DNA.

**Fig. 3.**
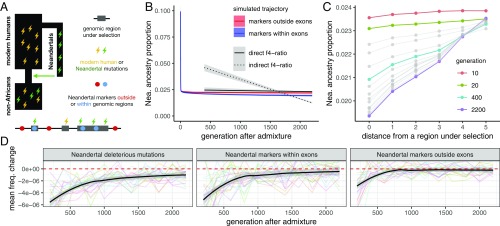
Simulations of selection against Neandertal ancestry. (*A*) Deleterious mutations (lightning bolts) accumulate in realistically distributed exonic sequence in modern humans and Neandertals. These regions accumulate additive, deleterious mutations, using a mutation rate of 10^−8^ per base pair per generation. To track the dynamics of Neandertal ancestry over time, neutral Neandertal markers are placed within (blue dots) and between (red dots) exons on all Neandertal chromosomes before introgression. (*B*) Simulated Neandertal ancestry proportions across 55 ky, in exonic and nonexonic sequence, averaged over 20 simulation replicates. Empirical observations from Fig. 1*A* are shown for comparison. Initial introgression levels were simulated at 10%. (*C*) Depletion of simulated Neandertal ancestry at neutral markers over time as a function of distance to regions under selection. Markers in bin 0 are those falling within exons; bins 1–5 represent quintiles of distance to the nearest exon. (*D*) Changes in frequencies of neutral Neandertal markers and deleterious Neandertal mutations over time, starting from generation 200. Each line shows average allele frequency changes over one simulation replicate. Black lines show smooth fits of these averages over 20 replicates.

Our observation that Neandertal ancestry levels did not significantly decrease from ∼45,000 y ago until today is seemingly at odds with the hypothesis that lower effective population sizes in Neandertals led to an accumulation of deleterious alleles, which were then subjected to negative selection in modern humans (3, 8–10). To investigate the expected long-term dynamics of selection against Neandertal introgression under this hypothesis, we simulated a model of the human genome with empirical distributions of functional regions and selection coefficients, extending a strategy previously applied by Harris and Nielsen (6). We simulated modern human and Neandertal demography, including a low long-term effective population size (*N*_e_) in Neandertals (Neandertal *N*_e_ = 1,000 vs. modern human *N*_e_ = 10,000) and 10% introgression at 55 kya (2,200 generations ago, assuming generation time of 25 y). To track the changes in Neandertal ancestry following introgression, we placed fixed Neandertal–human differences as neutral markers, both outside regions that accumulated deleterious mutations (to study the effect of negative selection on linked genome-wide neutral Neandertal variation) as well as within regions directly under selection (to track the effect of negative selection itself) (Fig. 3*A*).

Similar to Harris and Nielsen (6), we observed abrupt removal of Neandertal alleles from the modern human population during the first ∼10 generations after introgression, followed by quick stabilization of Neandertal ancestry levels (Fig. 3*B*). Compared with empirical estimates of Neandertal ancestry, we find a better fit between these simulations and the direct *f*_4_-ratio estimate than with the indirect *f*_4_-ratio estimate, suggesting that our direct Neandertal ancestry estimates are consistent with theoretical expectations of genome-wide selection against introgression (Fig. 3*B*). Specifically, simulations show −0.004% change in Neandertal ancestry over 45 ky; in the empirical data this slope is not rejected using the direct *f*_4_-ratio (*P* = 0.29), but is significantly different from the indirect *f*_4_-ratio (*P* < 0.001).

Because many factors can potentially influence the efficacy of negative selection, and no model fully captures all of these, we next sought to determine whether there is a combination of model parameters that could potentially lead to long-term continuous removal of Neandertal ancestry over time. Surprisingly, we failed to find a model which would produce a significant decline over time, although we tried by: (*i*) decreasing the long-term Neandertal *N*_e_ before introgression (making purifying selection in Neandertals even less efficient), (*ii*) increasing the *N*_e_ of modern humans after introgression (i.e., increasing the efficacy of selection against introgressed alleles), (*iii*) artificially increasing the deleteriousness of Neandertal variants after introgression (approximating a “hybrid incompatibility” scenario), (*iv*) simulating mixtures of dominance coefficients, or by (*v*) increasing the total amount of functional sequence (thereby increasing the number of accumulated deleterious variants in Neandertals and modern humans) (*SI Appendix*, Figs. S9–S13). Varying these factors primarily affected the magnitude of the initial removal of introgressed DNA by increasing the number of perfectly linked deleterious mutations in early Neandertal–modern human offspring (decreasing their fitness compared with individuals with less Neandertal ancestry), which in turn influenced the final level of Neandertal ancestry in the population (*SI Appendix*, Figs. S9–S13).

The depletion of Neandertal ancestry around functional genomic elements in modern human genomes has also been taken as evidence for selection against Neandertal introgressed DNA (3, 8). We next examined the genomic distribution of Neandertal markers at different time points in our simulations to determine whether our models can recapitulate these signals. In agreement with empirical results in present-day humans (3), we found a strong negative correlation between the proportion of Neandertal introgression surviving at a locus and distance to the nearest region under selection (Fig. 3*C*). Furthermore, we found that the strength of this correlation increases over time, with the bulk of these changes occurring between 10 and 400 generations postadmixture [mean Pearson’s correlation coefficient ρ = 0.07, 0.79, 0.96 at generations 10, 400, and 2,200, respectively (*SI Appendix*, Fig. S15)]. We note that this time period predates all existing ancient modern human sequences, frustrating any current comparison with empirical data. However, despite no apparent change in genome-wide Neandertal ancestry proportion over time, we observe a smaller though still significant decrease in linked Neandertal ancestry during the time period for which modern human sequences exist (∼400–2,200 generations post-admixture) (Fig. 3 *C* and *B*). Indeed, by looking at the average per-generation changes in frequencies of simulated Neandertal mutations (that is, derivatives of allele frequencies in each generation), we observe the impact of negative selection on linked neutral Neandertal markers until at least ∼700 generations post admixture (Fig. 3*D*) and find that it closely follows the pattern of introgressed deleterious mutations (Fig. 3*D*). After this period of gradual removal, selection against linked neutral variation slows down significantly as genome-wide Neandertal ancestry becomes largely unlinked from regions that are under negative selection (Fig. 3*D*). In contrast, the selected variants themselves are still removed, although at increasingly slower rates (Fig. 3*D*). Due to this slow rate, and the small contribution these alleles make to genome-wide Neandertal ancestry, their continued removal has little impact on the slope of Neandertal ancestry over time.

### Neandertal DNA Is Depleted in Regulatory and Conserved Noncoding Sequence.

**Fig. 4.**
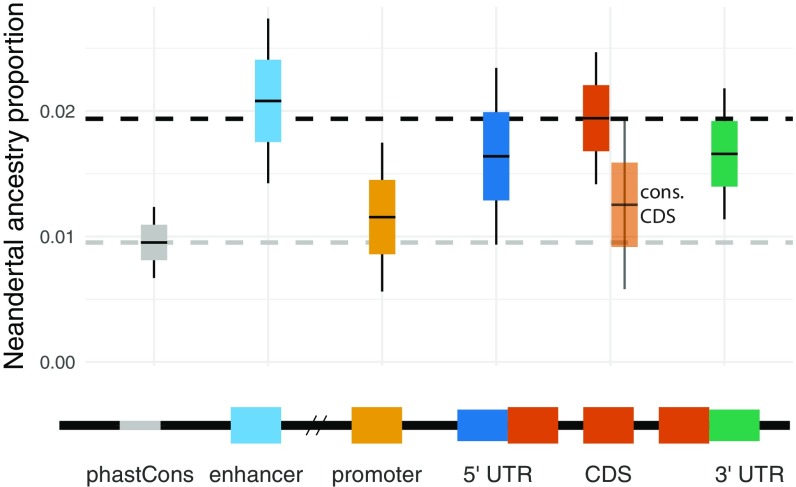
Neandertal ancestry estimates by genomic region. (*Top*) Direct *f*_4_-ratio estimates of Neandertal ancestry in all non-African SGDP individuals except Oceanians (known to carry Denisovan ancestry in addition to Neandertal ancestry) (25), with SNPs partitioned by functional annotation (Ensembl) or conservation (phastCons); “gap” combines intronic and intergenic sequence (dashed black line). Many annotation categories overlap other categories (*SI Appendix*, Table S1)—the largest is the 62% of protein-coding sequence which overlaps phastCons conserved elements (translucent orange). To minimize the noise in Neandertal ancestry estimates for small subsets of the genome, we calculated the direct *f*_4_-ratio using all SGDP Africans, except those that carry a high proportion of Neandertal alleles (Mozabite, Saharawi, Ju/’hoan North, Khomani San and Somali in *SI Appendix*, Fig. S4). Gray dashed line shows mean Neandertal ancestry in conserved phastCons regions. (*Bottom*) Idealized representation of genomic regions.

We next sought to leverage the direct *f*_4_-ratio in analyses of selection against introgression in functional genomic regions. Although previous studies have identified a depletion of Neandertal DNA in genomic regions with a high degree of evolutionary conservation, these studies have relied on maps of introgressed haplotypes (3, 29). Such maps may lack power to detect introgressed Neandertal DNA in highly conserved regions, as these regions may contain fewer informative sites carrying Neandertal–modern human differences. Furthermore, previous studies of negative selection against introgressed Neandertal DNA divided the genome into bins based on measures of evolutionary conservation, such as B values (30), which are not easily interpreted in terms of functional significance. To determine whether particular functional classes of genomic sites are differently affected by Neandertal introgression, we partitioned the human genome by functional annotation obtained from Ensembl v91 (31), and by primate conserved regions inferred using phastCons (32). For each annotation category, we estimated the Neandertal ancestry proportion in non-African Simons Genome Diversity Project (SGDP) individuals (excluding Oceanians) using the direct *f*_4_-ratio (Fig. 4).

In seeming contrast with previous studies (3, 8), we observed no significant depletion of Neandertal ancestry in CDS compared with intronic and intergenic regions (referred to as “gap” regions below) (average direct *f*_4_-ratio ∼1.94% in both; Fig. 4). However, we did identify a striking depletion of Neandertal ancestry in both promoters and phastCons conserved regions (1.15% and 0.95%), with both containing significantly less Neandertal ancestry than gap regions (*P* = 0.004 and *P* < 0.0001, estimated via resampling as described in *SI Appendix*, section S1). We note that 62% of CDS overlaps with phastCons regions (21% of phastCons conserved tracks overlap CDS); indeed, conserved CDS has a lower Neandertal ancestry estimate (1.25%) than overall CDS, although not as low as all phastCons regions (Fig. 4). These results suggest that previously observed depletions in conserved and genic regions may not have been driven primarily by protein-coding differences between Neandertals and modern humans, as was previously assumed, but rather by differences in promoters and other noncoding conserved sequence. This hypothesis is supported by several recent studies of the effects of introgressed Neandertal sequences, including those with signatures of adaptive introgression, which found that surviving functional introgressed haplotypes have their major influence on gene expression regulation (33–37).

We note that the lack of a depletion in CDS does not fit the observations from our simulations (Fig. 3*C*). Assuming additivity, and a distribution of fitness effects (DFEs) derived from the frequency spectra of mutations altering coding sequence (38), these simulations predict a reduction of 5–17% Neandertal ancestry versus nonselected regions, depending on distance from selected regions (Fig. 3*C*). In addition, the reduction in simulations is much smaller than the empirical depletions of promoter and phastCons regions (40% and 51%, respectively). Together, these demonstrate that the actions of selection against Neandertal sequence are not fully captured by the models presented here. Although it is beyond the scope of this work, it may be possible to leverage distributions of Neandertal ancestry in studying the action of selection in noncoding sequence. Challenges associated with such work include the uncertainty of the DFE of mutations affecting noncoding sequence, and their dominance coefficients, potential epistatic effects of regulatory mutations, as well as the fact that a single deleterious mutation can affect a region falling into multiple functional categories at once (*SI Appendix*, Table S1).

## Conclusions

Our reevaluation of Neandertal ancestry in modern human genomes indicates that overall levels of Neandertal ancestry in Europe have not significantly decreased over the past 45,000 y, and that previous observations of continuous Neandertal ancestry decline were likely an artifact of unaccounted-for gene flow increasing allele sharing between West Eurasian and African populations. Nevertheless, we do find evidence of selection against Neandertal DNA in the genome-wide distribution of Neandertal ancestry, with such ancestry depleted in promoter and other noncoding conserved DNA more strongly than in protein-coding sequence, raising the possibility that Neandertals may have differed more from modern humans in their regulatory variants than in their protein-coding sequences, and that regulatory variation may provide a richer template for selection to act upon.

Furthermore, simulations suggest that negative selection against introgression is expected to have the strongest impact on genome-wide Neandertal ancestry during the first few hundred generations, before the time frame for which ancient samples are currently available. The genomes of early modern humans living 55–50 kya, although difficult to obtain, may shed additional light on the process of selection against Neandertal DNA, as well as on early out-of-Africa demography.

Our findings can be extrapolated to other cases where one species or population contributes a fraction of ancestry to another species or population, a frequent occurrence in nature (5, 29, 39–41). Even in cases where the introgressing population carries a high burden of deleterious mutations, negative selection is not expected to result in an extended decrease in the overall genome-wide ancestry contributed by that population. Therefore, any long-term shifts in overall ancestry proportions over time are likely to be the result of forces other than negative selection, for example admixture with one or more other populations.

## Materials and Methods

### Source Code and Jupyter Notebooks.

Complete source code for data processing and simulation pipelines, as well as R and Python Jupyter notebooks with all analyses, can be downloaded from the project repository on GitHub: https://www.github.com/bodkan/nea-over-time.

### Data Processing.

SNP data captured at ∼2.2 million loci from a set of Upper Paleolithic individuals published by Fu et al. (8) were obtained from the David Reich laboratory (https://reich.hms.harvard.edu/datasets), and merged with previously published genotypes for the Altai Neandertal (23), Vindija Neandertal (13), Denisovan (42), and SGDP (25) to create a single EIGENSTRAT dataset. For all analyses, individuals with at least 200,000 captured sites were analyzed. SNP data captured using the “archaic admixture array” (SNP panel 4 in ref. 22) published by Fu et al. (8) were also downloaded from the Reich laboratory website and filtered to sites homozygous in the Altai and Vindija Neandertal genomes, resulting in a set of ∼480,000 sites carrying nearly fixed Yoruba–Neandertal differences.

### Admixture Statistics.

All *f*_4_ statistics, f_4_-ratio, and qpAdm statistics were calculated on the merged 2.2 million loci EIGENSTRAT dataset using our R package admixr (available from https://www.github.com/bodkan/admixr) which utilizes the ADMIXTOOLS software suite for all underlying calculations (15).

### Estimates of Neandertal Ancestry.

Indirect *f*_4_-ratio estimates (Fig. 1*A*, dashed line) were calculated as 1 − *f*_4_(West and Central Africans, Chimpanzee; *X*, Archaics)/*f*_4_(West and Central Africans, Chimpanzee; East African, Archaics), where West and Central Africans are Yoruba, Mbuti, and Mende from the SGDP panel, East Africans are SGDP Dinka, and archaics are the Altai Neandertal (23) and Denisovan (42) individuals (*SI Appendix*, Fig. S1), as described in the original Fu et al. study (8). Direct *f*_4_-ratio estimates (Fig. 1*A*, solid line) were calculated as *f*_4_(Altai, Chimpanzee; *X*, African)/*f*_4_(Altai, Chimpanzee; Vindija, African) (*SI Appendix*, Fig. S1). Neandertal ancestry proportions using qpAdm were estimated assuming a two-source model, with the Vindija Neandertal and Mbuti as potential sources, and Chimpanzee, the Altai Neandertal, and the Denisovan as outgroups. Admixture array-based Neandertal ancestry estimates were calculated as the proportion of alleles in a test individual matching the allele seen in Neandertals. Confidence intervals and *P* values were calculated using a resampling strategy described in *SI Appendix*, section S1.

### Affinity of Ancient and Present-Day Individuals Toward Africans over Time.

We calculated *f*_4_ statistics in the form *f*_4_(Ust’-Ishim, *X*; *Y*, Chimpanzee), which test for changes in the sharing of derived alleles between a series of West Eurasians (*X*) and population *Y* with respect to Ust’-Ishim, an ancient hunter-gatherer that predates the split of West and East Eurasians (43) (*SI Appendix*, Fig. S2). Admixture between *X* and *Y* or populations related to *X* and *Y* is expected to lead to an increase in the proportion of shared derived alleles.

### Testing for the Presence of Basal Eurasian Ancestry.

We used the statistic *f*_4_(West Eurasian *W*, Han; Ust’-Ishim, Chimpanzee) to look for evidence of Basal Eurasian ancestry in a West Eurasian *W* (*SI Appendix*, Fig. S4) (28). This statistic tests if the data are consistent with a tree in which *W* and Han lineages form a clade, which results in *f*_4_ statistic not significantly different from 0. Significantly negative values are evidence for an affinity between the Ust’-Ishim and Han lineages, which could be explained by *W* carrying ancestry from a population that diverged from the non-African lineage before the split of Ust’-Ishim.

### Neutral Coalescent Simulations.

To study the effects of gene flow between non-African and African populations on various admixture statistics, we simulated different scenarios of such gene flow using a neutral coalescent programming library, msprime (44) (*SI Appendix*, Fig. S8). Depending on the particular analysis (Fig. 2 and *SI Appendix*, Fig. S2 and S3), we calculated admixture statistics (*f*_4_, *f*_4_-ratio, and admixture array proportions) as described above using SNPs extracted from each simulation run. Detailed description of the simulations can be found in *SI Appendix*, section S2.

### Simulations of Selection.

To study the dynamics of selection against Neandertal introgression over time, we used the simulation framework SLiM 2 (45) to build a realistic model of the human genome with empirical distributions of functional and conserved regions and selection coefficients, extending and generalizing a strategy previously applied by Harris and Nielsen (6) (Fig. 3*A*). First, we simulated a demography of modern humans and Neandertals (low long-term *N*_e_) before the introgression, and let the simulated genomes accumulate deleterious mutations. Then we simulated a single pulse of admixture from Neandertals into the non-African population at a rate of 10% and tracked the changes in Neandertal ancestry in an admixed population at fixed neutral Neandertal markers distributed along each Neandertal genome before the introgression. A detailed description of our simulations and analyses of simulated data can be found in *SI Appendixes*, sections S3 and S4.

## Supplementary Material

Supplementary File
