# Supplementary material for: Limits of long-term selection against Neandertal introgression
Source: Proc Natl Acad Sci U S A. 2019 Jan 15;116(5):1639–44. doi: 10.1073/pnas.1814338116 (PMC6358679; doi:10.1073/pnas.1814338116)
Supplement: Supplementary File [file pnas.1814338116.sapp.pdf]

PNAS
www.pnas.org

Supplementary Information for

Limits of long-term selection against Neandertal introgression

Martin Petr[1], Svante Pääbo[1], Janet Kelso[1]*, Benjamin Vernot[1]*

[1] Department of Evolutionary Genetics, Max Planck Institute for Evolutionary Anthropology, Deutscher Platz 6, 04103, Leipzig, Germany.
* Contributed equally / corresponding author

Email: kelso@eva.mpg.de benjamin_vernot@eva.mpg.de

This PDF file includes:

Supplementary Information Text
Figs. S1 to S15
Table S1
References for SI reference citations

www.pnas.org/cgi/doi/10.1073/pnas.1814338116

Supplementary Information Text

S1. Neandertal ancestry estimate confidence intervals and *p*-values

Confidence intervals on the slope of time vs Neandertal ancestry proportion were calculated empirically via resampling. For each individual, we sampled 10,000 Neandertal ancestry estimates from a normal distribution centered on the true estimate, with standard deviation equal to the standard error provided by ADMIXTOOLS. We then fit 10,000 linear models, extracted the 95% confidence intervals across all 10,000 resulting slopes. From these 10,000 slopes we can also calculate an empirical *p*-value for any given slope (generally for a slope of 0, but -0.004 for the comparison of simulated data vs the direct and indirect f_4-ratio estimates). For comparisons of the ratio of Neandertal ancestry between populations (e.g., individuals with and without Basal Eurasian ancestry), we similarly sampled 10,000 Neandertal ancestry estimates, and calculated an empirical *p*-value for a ratio of 1 between the two groups. For a comparison of functional annotation categories, we similarly resampled 10,000 Neandertal estimates from a given category, and calculated an empirical *p*-value that these resamplings reject the Neandertal ancestry proportion calculated for gap regions.

S2. Simulations of gene flow between non-Africans and Africans

We simulated different scenarios of gene flow between Africans and non-Africans after Neandertal introgression using the neutral coalescent programming library *msprime* (1) (Fig. S8). We used the following demographic parameters: split of a chimpanzee lineage at 6 million years ago, split of Neandertals from anatomically modern humans at 500 kya, split within Africa at 150 kya, and split of non-Africans from one of the two African lineages at 60 kya with a 5 ky long bottleneck of N_e = 2000. We simulated a single 3% pulse of Neandertal admixture into a constant-size non-African population at 55 kya. We sampled one chimpanzee chromosome, 4 Neandertal chromosomes sampled at 80 kya, single chromosomes from the non-African lineage sampled at regular time intervals over the time range of Upper-Paleolithic and present-day individuals from our data, and two pairs of chromosomes from the two present-day African populations. We simulated 500 replicates of 100 Mb chromosomes (Fig. S2) or 600 replicates of 0.25 Mb chromosomes

(Fig. 2, S3) using a mutation rate of 1×10^{-8} mutations per bp per generation and a recombination rate of 1×10^{-8} crossovers per bp per generation, and converted them into tables of all simulated SNPs for easier calculation of admixture statistics. For analysis of the "admixture array", we also generated a second set of SNPs by filtering only for sites carrying fixed African-Neandertal differences (to approximate the ascertainment of the archaic admixture array – SNP panel 4 in (2)). To estimate the true Neandertal ancestry levels we examined the origin of each simulated mutation in *msprime* and extracted only those SNPs that truly originated in the Neandertal population. Using this set of sites avoids any issues caused by introduction of Neandertal alleles into Africans via gene-flow from admixed non-Africans.

In Fig. 2 and Fig. S3, we evaluated the behavior of the admixture array ancestry proportion and direct and indirect f_4-ratio estimates under three scenarios: (i) no gene flow between Africans and non-Africans post Neandertal admixture, (ii) gene flow from non-Africans into both African populations, (iii) gene flow from one African population into non-Africans, (iv) bi-directional gene flow between Africans and non-Africans.

Using the simulated SNP sets (all SNPs and archaic admixture array-like set), we calculated direct and indirect f_4-ratio estimates, as well as admixture array proportion estimates, as described above. Unbiased levels of Neandertal ancestry were calculated on the set of true Neandertal-derived SNPs. As the statistics can be relatively noisy, we calculated average values of each individual statistic over all simulation replicates.

S3. Simulations of selection

We used the simulation framework *SLiM 2* (3) to build a realistic model of the human genome with empirical distributions of functional regions and selection coefficients, extending and generalizing a strategy previously applied by Harris and Nielsen (4). To obtain the coordinates of regions under negative selection, we downloaded the positions of different classes of annotated genomic regions from the Ensembl database (5) (Ensembl Genes 91 and Ensembl Regulation 91) and conserved regions from the phastConsElements46wayPrimates track from the UCSC Genome Browser (6, 7) (updated 2009-11-21). In each simulation, we encoded those regions in a genomic structure in *SLiM*'s *Eidos* programming language, maintaining the distances between

them. In order to model the heterogeneity of recombination rate along a genome in our simulations, we used empirically estimated genetic distances between all simulated genomic features using a recombination map inferred by the HapMap project (http://hapmap.ncbi.nlm.nih.gov/downloads/recombination/2011-01_phaseII_B37/) (8). To approximate a mixture of strongly, weakly and nearly-neutral deleterious mutations, we used a distribution of fitness effects (DFE) estimated from the frequency spectrum of human non-synonymous mutations (9). The rate of accumulation of new mutations was set to $1x10^{-8}$ per bp per generation.

The simulations themselves were performed in two steps (Fig. 3A), using a combination of human and Neandertal demographic models used in previous introgression studies (4, 10). In the first step, we simulated a simplified demography of modern humans and Neandertals prior to the introgression, starting with a burn-in period of 70,000 generations, to let the simulated genomes with mutations reach an equilibrium state (the length of this burn-in period was determined as 7 * ancestral human N_e, which was therefore set to a constant 10,000). The split of Neandertals and modern humans was set to 500,000 years ago, with N_e of Neandertals and modern humans set to constant values of 1,000 and 10,000, respectively. This burn-in period was performed for each specific simulation scenario separately. At the end of the burn-in step, we simulated the split of African and non-African populations at 55 kya. Following the split, the non-African population experienced a bottleneck with N_e = 1861 (as inferred by Gravel et al. (11)). All simulated individuals and accumulated mutations were saved to a population output file for use in the second step.

In the second step, we simulated a single pulse of admixture from Neandertals into the non-African population at a rate of 10%. To track Neandertal ancestry along simulated genomes through time, we placed 50,000 neutral Neandertal markers outside of any potentially functional sequence (which was determined as a union of all annotated Ensembl regions) (Fig. 3A). The locations of these markers were randomly sampled from the set of nearly-fixed Yoruba-Neandertal differences present on the archaic admixture array (SNP panel 4 in (2)). Furthermore, to be able to track Neandertal ancestry within regions directly under negative selection, we placed additional set of fixed Neandertal markers within those regions (Fig. 3A).

Because the efficacy of selection is related to the N_e of the population under consideration (12), we evaluated different demographic models for non-Africans, including a widely-used model by Gravel et al. (11) (i.e. long bottleneck followed by a period of exponential growth), a model of initial slow linear growth post admixture, as well as a model of constant N_e after Neandertal introgression (Fig. S14). However, because we found that the N_e of the admixed non-African population did not have an impact on the slope of the trajectory of Neandertal ancestry over time (Fig. S10), the main results in our paper were performed using a demographic model with constant N_e = 10,000.

To track dynamics of selection over time, we periodically saved the simulation state, saving all mutations still segregating at each time-point (both neutral markers and deleterious modern human and Neandertal mutations) in a sample of 500 diploid individuals in VCF format for further analysis. For efficiency reasons, we saved only simulation states in generations 1-10, 20, 50, 100 and then every 200th generation until the final generation 2200 (i.e. 55 thousand years post-introgression, assuming generation time of 25 years).

S4. Evaluating the effect of negative selection against introgression

All of the following analyses were performed on VCF outputs from 20 replicates of our *SLiM* simulations, described in the previous section. Trajectories of Neandertal ancestry in a population over time (Figs. 3B and S9-13) were calculated by averaging the frequencies of all neutral Neandertal markers in a simulation in each time point across 500 sampled diploid individuals. Analysis of the efficacy of selection against introgression as a function of distance from regions carrying deleterious variants (Fig. 3C) was performed by binning the 50,000 neutral Neandertal markers into 5 quintiles, based on their distance from the nearest region under selection. The lowest bin "0" contains neutral Neandertal markers within regions that carried accumulated deleterious mutations. Neandertal ancestry proportions were then calculated for each of the 1,000 sampled chromosomes in each bin, combined from all 20 simulation replicates. Analysis of allele frequency changes over time was performed by calculating the frequency change of each class of variant (neutral Neandertal markers within and outside of selected

regions, and deleterious mutations) between each consecutive pair of sampled time-points, and then averaged over all mutations. For example, if x and y are allele frequencies of a mutation at time-points a and b, then the allele frequency change was calculated as $(x - y) / (a - b)$. This calculation was repeated for all 20 simulation replicates and mean frequency changes were plotted for each replicate separately (Fig. 3D).

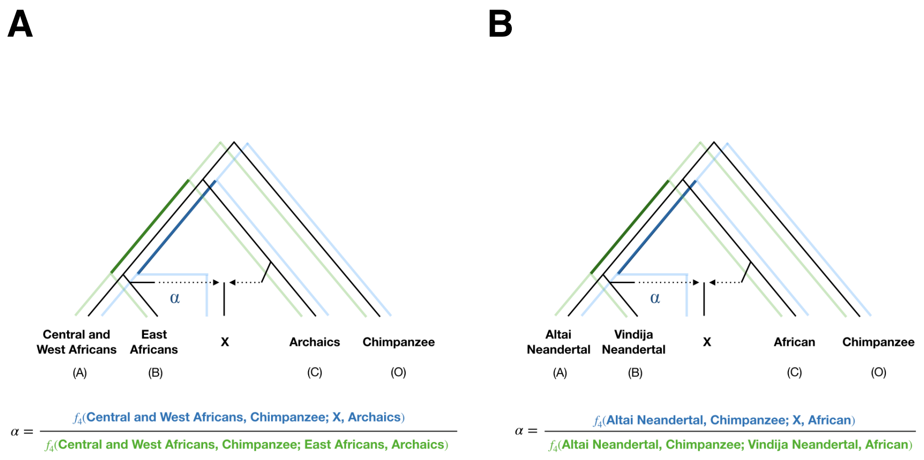

Fig. S1. Tree models underlying indirect and direct f4-ratio Neandertal ancestry estimates.

A: Tree model used for the indirect f_4-ratio. **B:** Tree model used for the direct f_4-ratio, utilizing two high coverage Neandertal genomes. Blue and green lines represent overlaps of drift paths of f_4 statistics in the numerator and the denominator of f_4-ratios (13, 14).

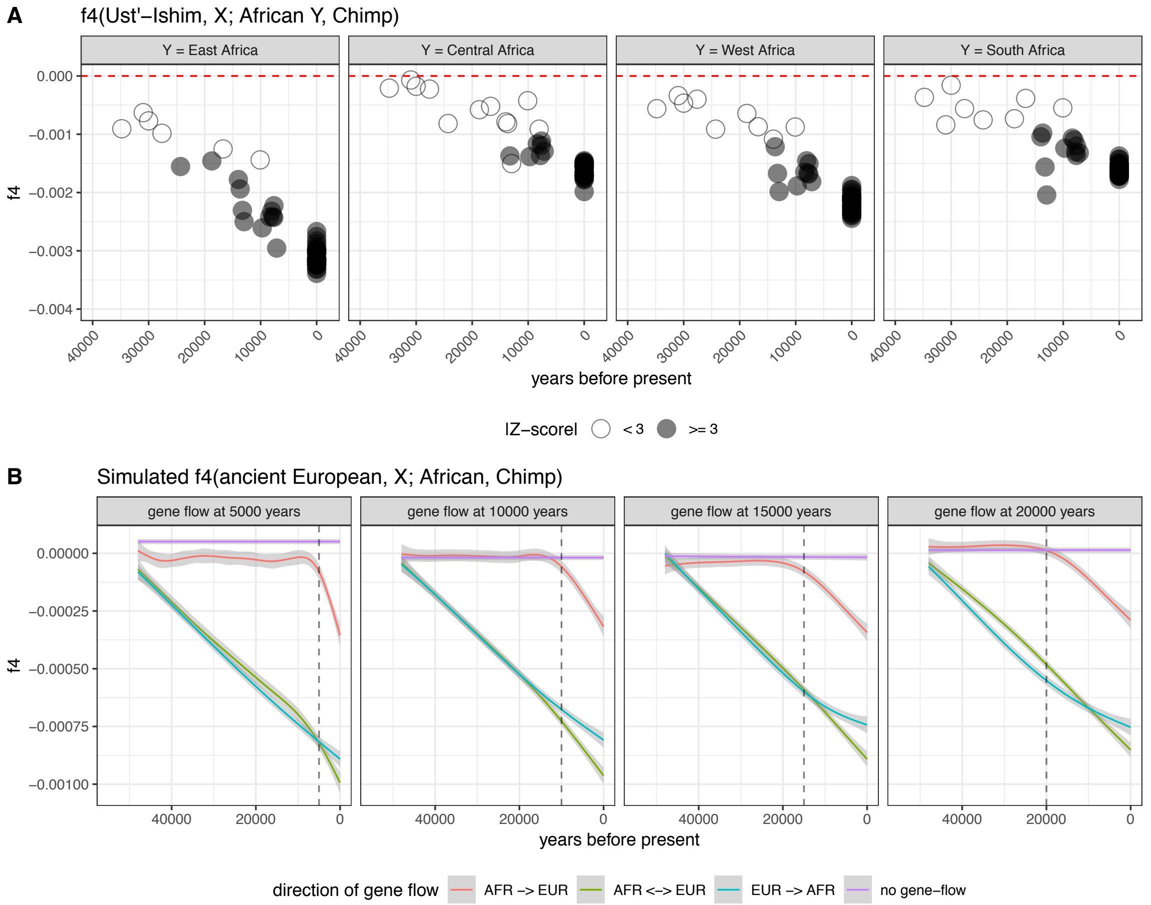

Fig. S2. Increasing affinity between West Eurasians and Africans over time.

A: The statistic *f4*(Ust'-Ishim, West Eurasian; African Y, Chimp) is increasingly negative over time for ancient and present-day West Eurasians (WE), indicating increasing allele sharing (affinity) between WE and Africans with respect to Ust'-Ishim. A variety of demographic forces could cause such shifts, including migration between West Eurasia and Africa, or migration of a third population into both West Eurasia and Africa. The f_4 statistic is not expected to be different from 0 in the absence of admixture. East Africans: Dinka, Bantu, Luhya, Luo, Masai, Somali, West Africans: Esan, Gambian, Mandenka, Mende, Yoruba, Central Africans: Mbuti, Biaka, South Africans: Khomani San, Ju|'hoan. **B:** Simulations of migration between West Eurasians and Africans starting from 5000 (left) to 20,000 (right) years ago, assuming N_e = 20000 in Africans, N_e = 5000 in West Eurasians and "total migration" gm = 0.1 (g is the duration of gene flow in generations, m is the proportion of the target population composed of migrants in each generation) show

that under a model of migration from West Eurasia to Africa (blue line), this *f4* statistic grows increasingly negative over the past 45ky, regardless of the when the migration event took place, thus making it difficult to determine the timing of such an event.

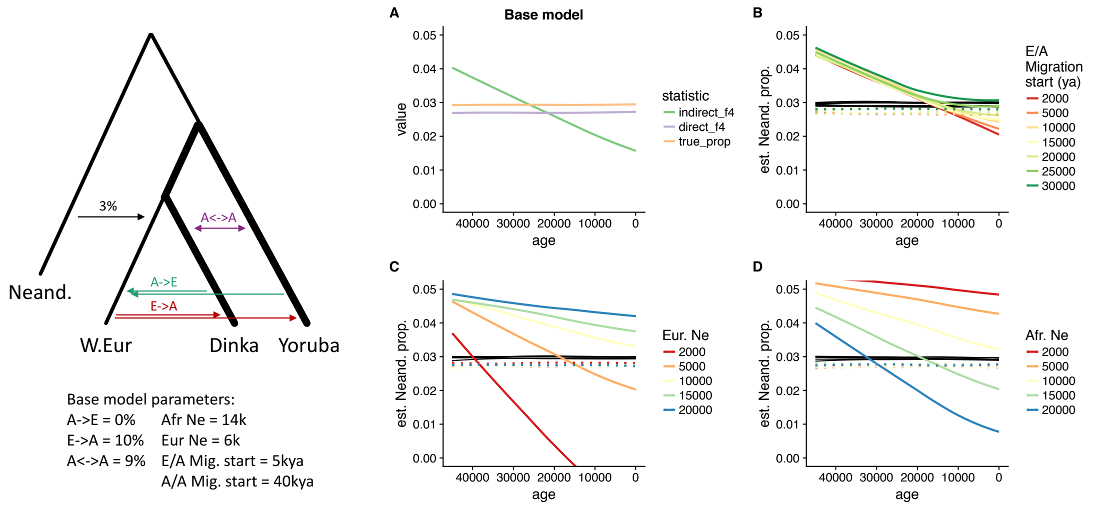

Fig. S3. The effect of Ne and timing on the patterns observed in indirect and direct f_4-ratio statistics.

A: True Neandertal ancestry, along with direct and indirect f_4-ratio estimates, on simulated neutral data according a single demographic model (left) with migration from Europe to Africa, and between two African populations (represented here as Dinka and Yoruba). **B:** Starting from the model in A, the effect of varying the timing of migration on the indirect (solid colored lines), direct (dotted lines), and true Neandertal ancestry proportions (solid black lines). **C)** Similarly, the effect of varying European N_e, and **D)** the effect of varying N_e in both African populations.

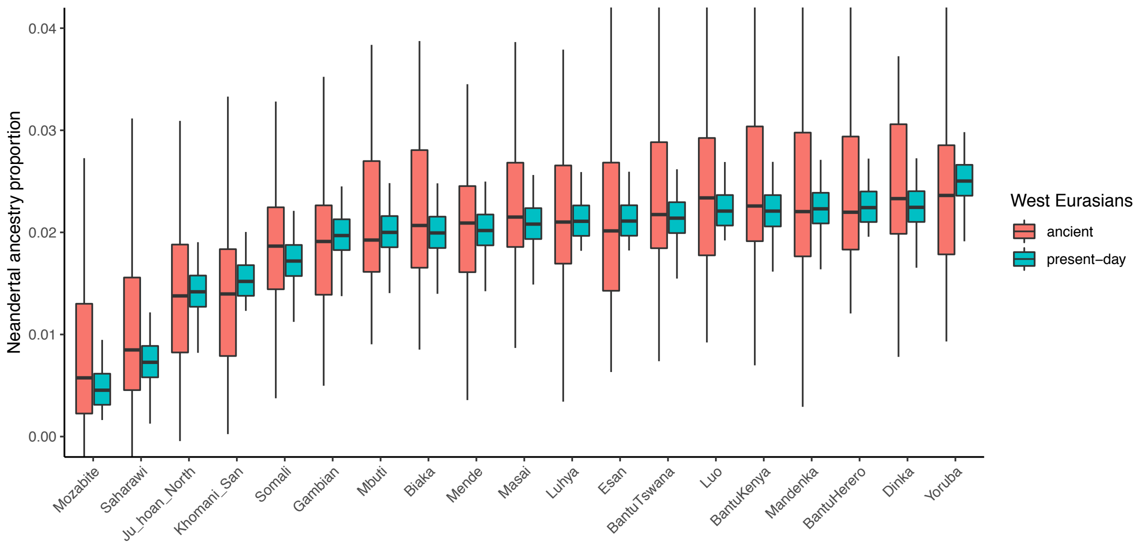

Fig. S4. Neandertal ancestry in ancient and present-day West Eurasians estimated with the direct f_4-ratio using various African populations.

Neandertal ancestry estimates in ancient and present-day West Eurasians were calculated using the direct f_4-ratio as f_4*(Altai Neandertal, Chimp; X, African) /* f_4*(Altai Neandertal, Chimp; Vindija Neandertal, African)* (Fig. S1). As shown with simulations in Fig. 2, in this statistic, the presence of Neandertal alleles in both Africans and *X* will cause an underestimate of the true Neandertal ancestry in *X*, which can be seen in these empirical estimates as well.

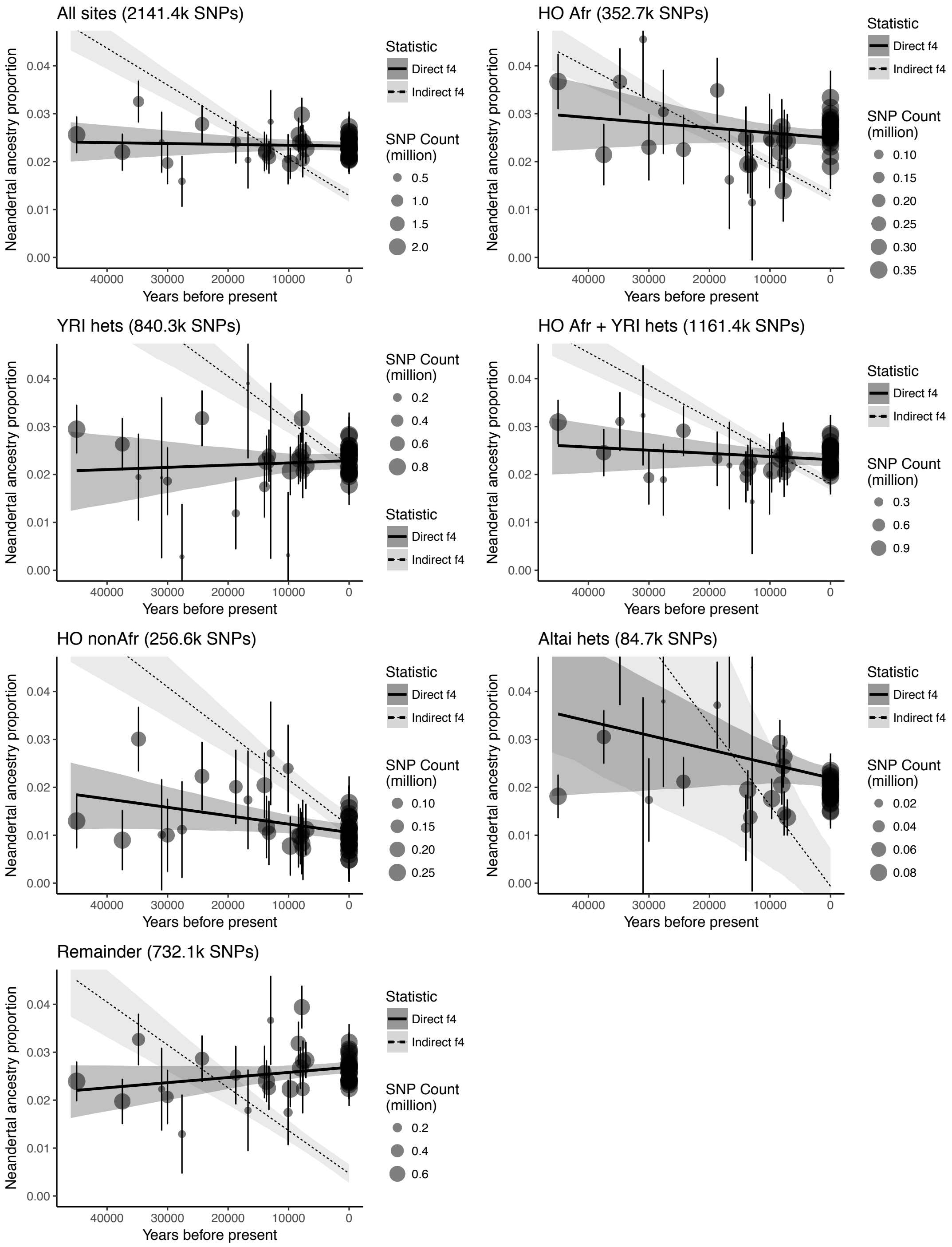

Fig. S5. f4-ratio calculations on ascertainment subsets.

Direct and indirect *f4*-ratios are calculated in the same manner as Figure 1, with the data partitioned according to seven ascertainment schemes (from left to right, top to bottom): All 2.2 million SNPs; African ascertained SNPs from the Human Origins array (HO);

Heterozygotes from two Yoruban individuals (YRI hets); Combined African ascertainments (HO nonAfr+ YRI hets); non-African ascertained SNPs from the Human Origins array (HO nonAfr); Heterozygotes in the Altai Neandertal (Altai hets); and the remaining 732k SNPs that do not fit into one of the previous categories. These "remaining" SNPs are largely from the Illumina 610-Quad array and Affymetrix 50k array. Details of these ascertainments can be found in (2).

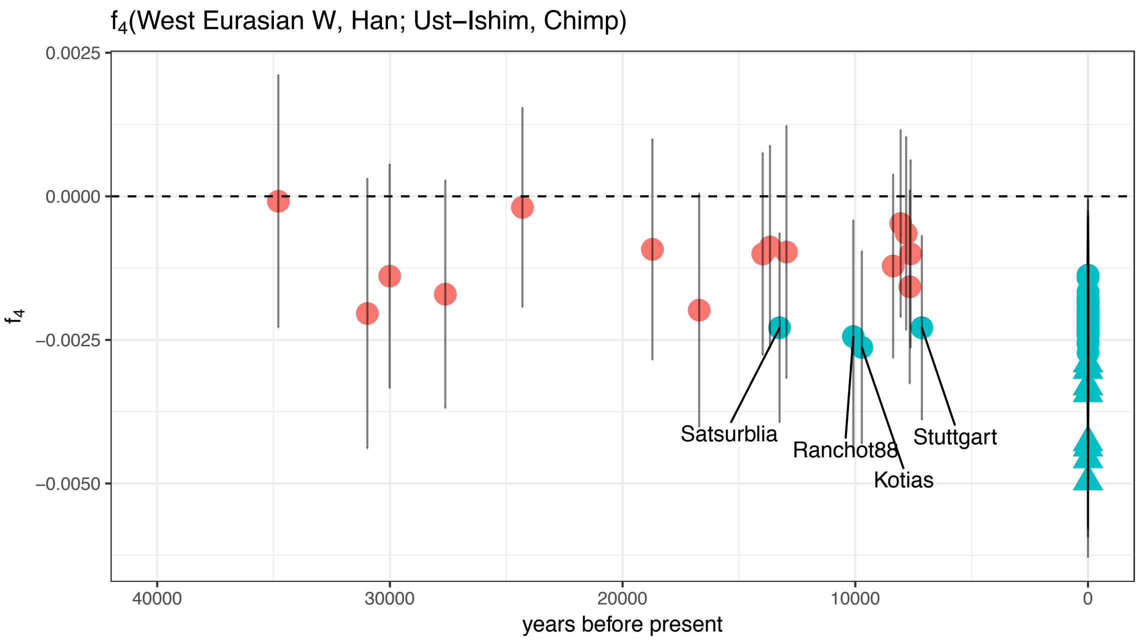

Fig. S6. A signal of Basal Eurasian ancestry in West Eurasia over time.

The statistic *f_4(West Eurasian W, Han; Ust'-Ishim, Chimp)* has been previously used as a test of the presence of Basal Eurasian ancestry in a West Eurasian *W* (15). Specifically, it tests whether a population tree in which *W* and Han lineages form a clade is consistent with the observed data, which results in f_4 statistic ~0. On the other hand, significantly negative values are evidence for an affinity of Han and Ust'-Ishim lineages, which can be most parsimoniously explained by *W* carrying an ancestry component from a population that diverged from other Eurasians prior to the separation of Ust'-Ishim. This "ghost" population is commonly referred to as Basal Eurasians (16). By analyzing a combined early-modern and present-day West Eurasian dataset, we find that this f_4 statistic becomes consistently negative in the present, which is in agreement with the hypothesis that present-day West Eurasians carry (in different proportions) Basal Eurasian ancestry that was not present in early European hunter gatherers. Blue color indicates individuals with significantly negative f_4 statistic. Present-day individuals are Europeans (circles) and Near Easterners (triangles) from the SGDP panel (17). The SGDP identifiers for Near East individuals used for this grouping are BedouinB, Yemenite_Jew, Palestinian, Iraqi_Jew, Jordanian, Druze, Iranian, Samaritan.

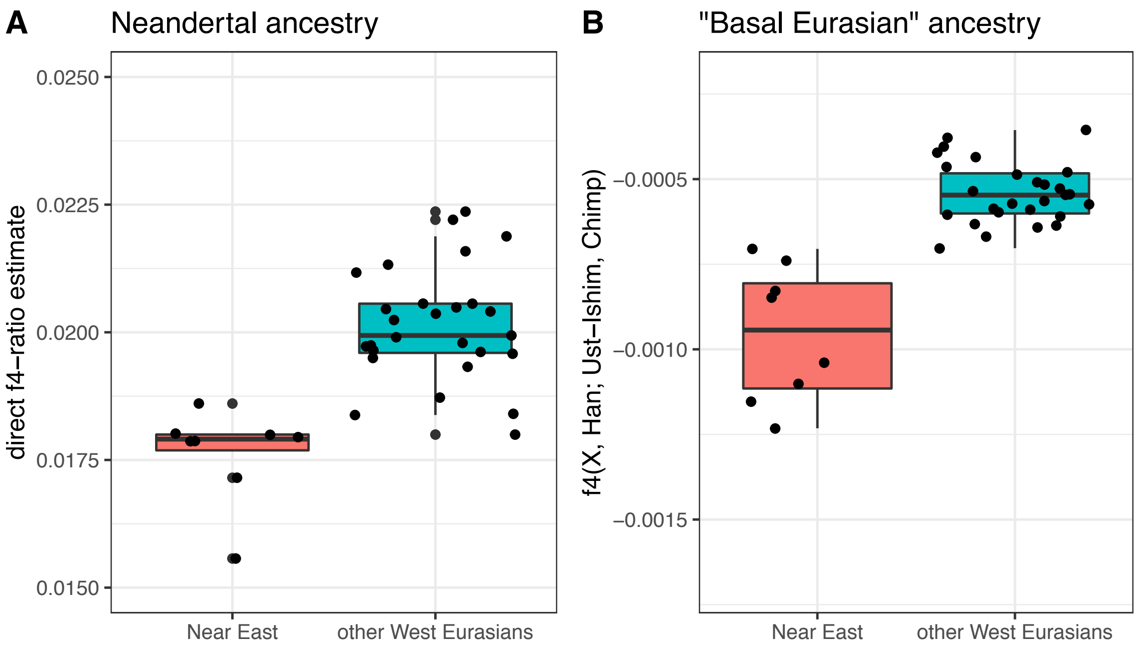

Fig. S7. Proportions of Neandertal ancestry (A) and the amounts of "Basal Eurasian" ancestry (B) in present-day Near Easterners vs other West Eurasians. Panel B shows the same data as the present-day data points in Fig. S6, but is split into two groups – Near Easterners and other West Eurasians. The SGDP identifiers for Near East individuals used for this grouping are BedouinB, Yemenite Jew, Palestinian, Iraqi Jew, Jordanian, Druze, Iranian and Samaritan (17).

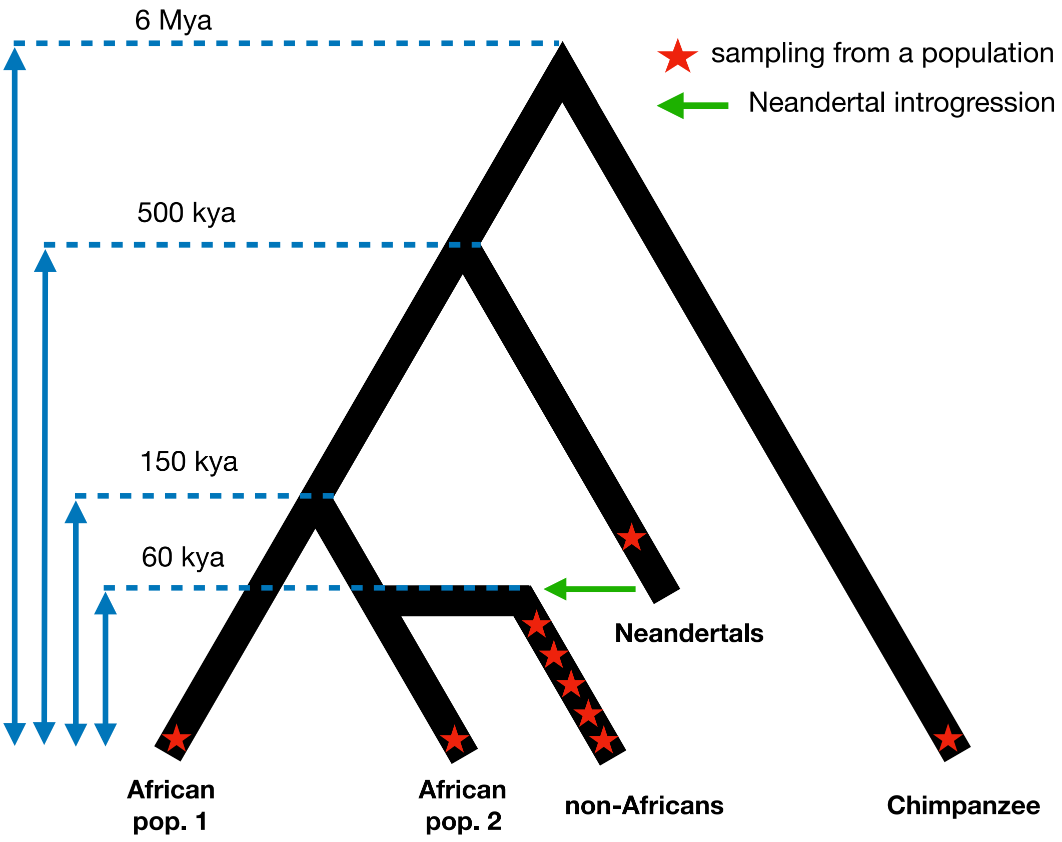

Fig. S8. Demographic model used for testing the temporal behavior of admixture statistics.

Blue dashed lines show split times between simulated populations, red stars indicate approximate points in time at which simulated chromosomes were sampled.

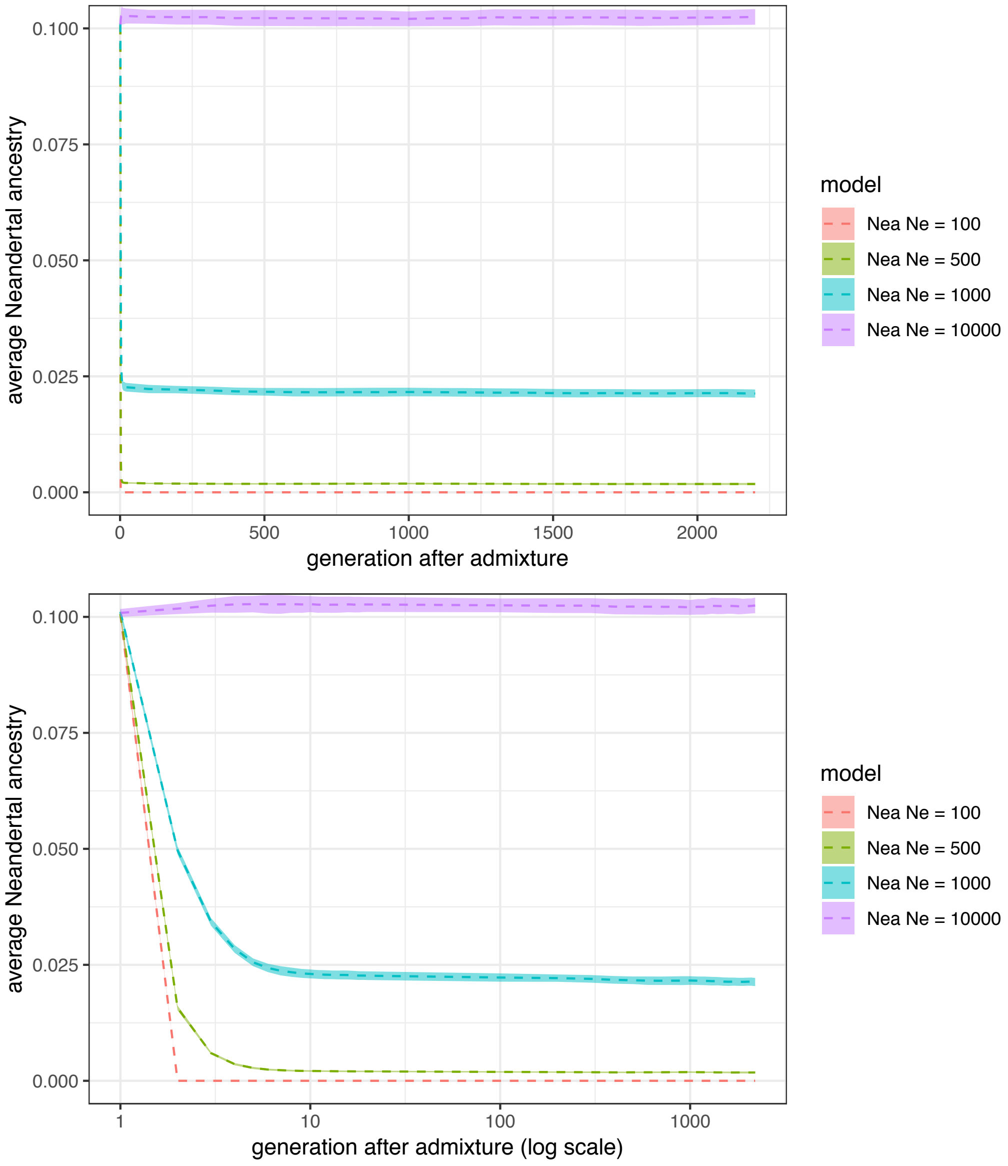

Fig. S9. The effect of Neandertal N_e (Nea *Ne*) on trajectories of Neandertal ancestry after introgression.

Top and bottom, panels show linear and logarithmic timescales, respectively. The lower the N_e of Neandertal population, the more deleterious alleles behave nearly neutrally, allowing them to reach high frequencies in the Neandertals (4, 18). This imposes a stronger genetic load of the initial modern-human-Neandertal hybrids, causing a more abrupt removal of Neandertal ancestry in the generations shortly after admixture. The shaded areas represent 95% confidence intervals.

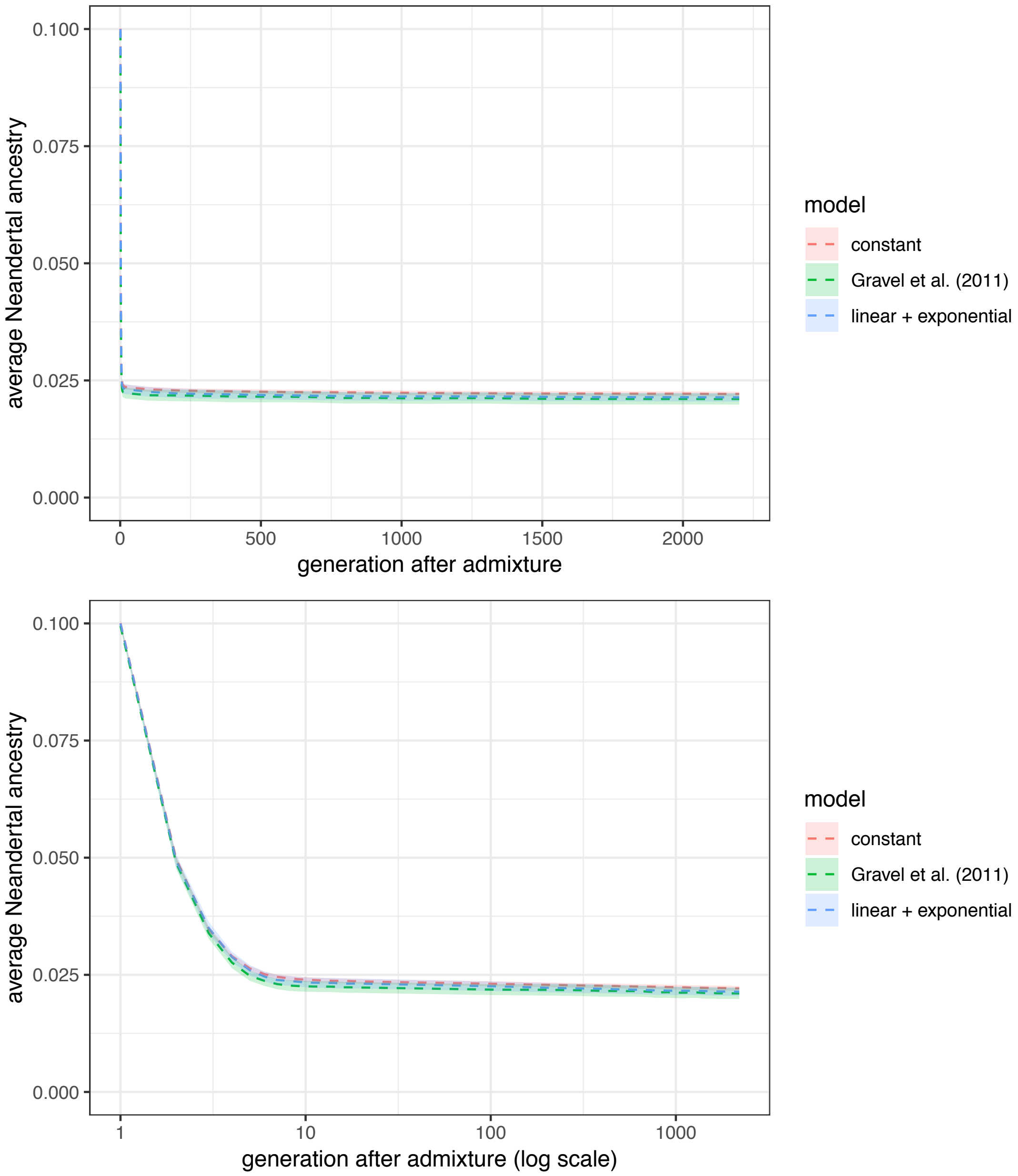

Fig. S10. The effect of non-African demography on trajectories of Neandertal ancestry after introgression.

Top and bottom panels show linear and logarithmic timescales, respectively. Although N_e as a function of time differs dramatically between all three demographic models that we considered (Fig. S14), changing this parameter does not have a strong impact on the overall shape of Neandertal ancestry trajectories. The shaded areas represent 95% confidence intervals.

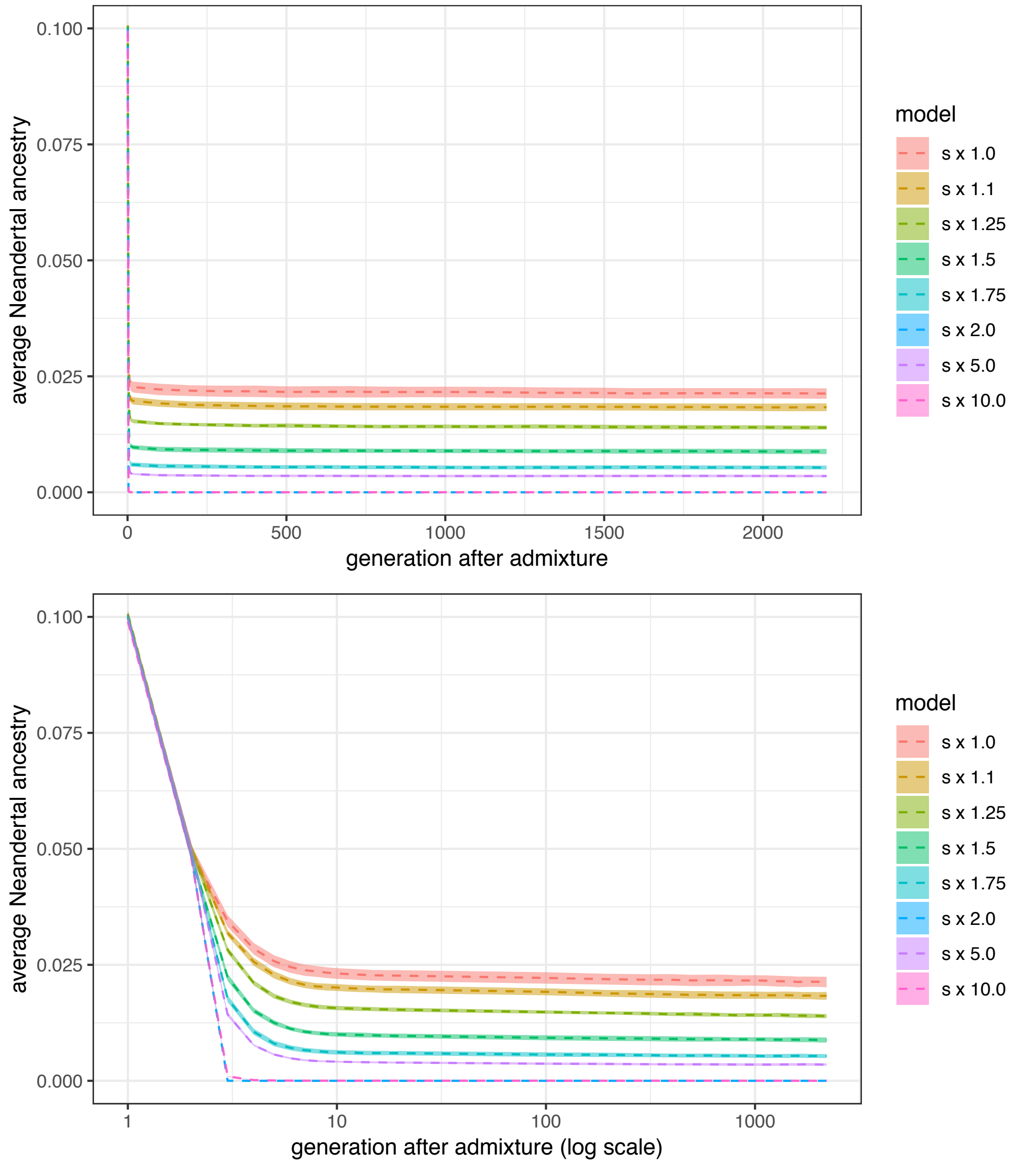

Fig. S11. The effect of making Neandertal mutations more deleterious by increasing their selection coefficients.

Top and bottom panels show linear and logarithmic timescales, respectively. We artificially increased the selection coefficient *s* of introgressed Neandertal deleterious mutations by multiplying their *s* by a constant factor. We find that this affects only the final level of Neandertal ancestry in the population, due to stronger genetic burden on hybrids in the first generations after admixture. The shaded areas represent 95% confidence intervals.

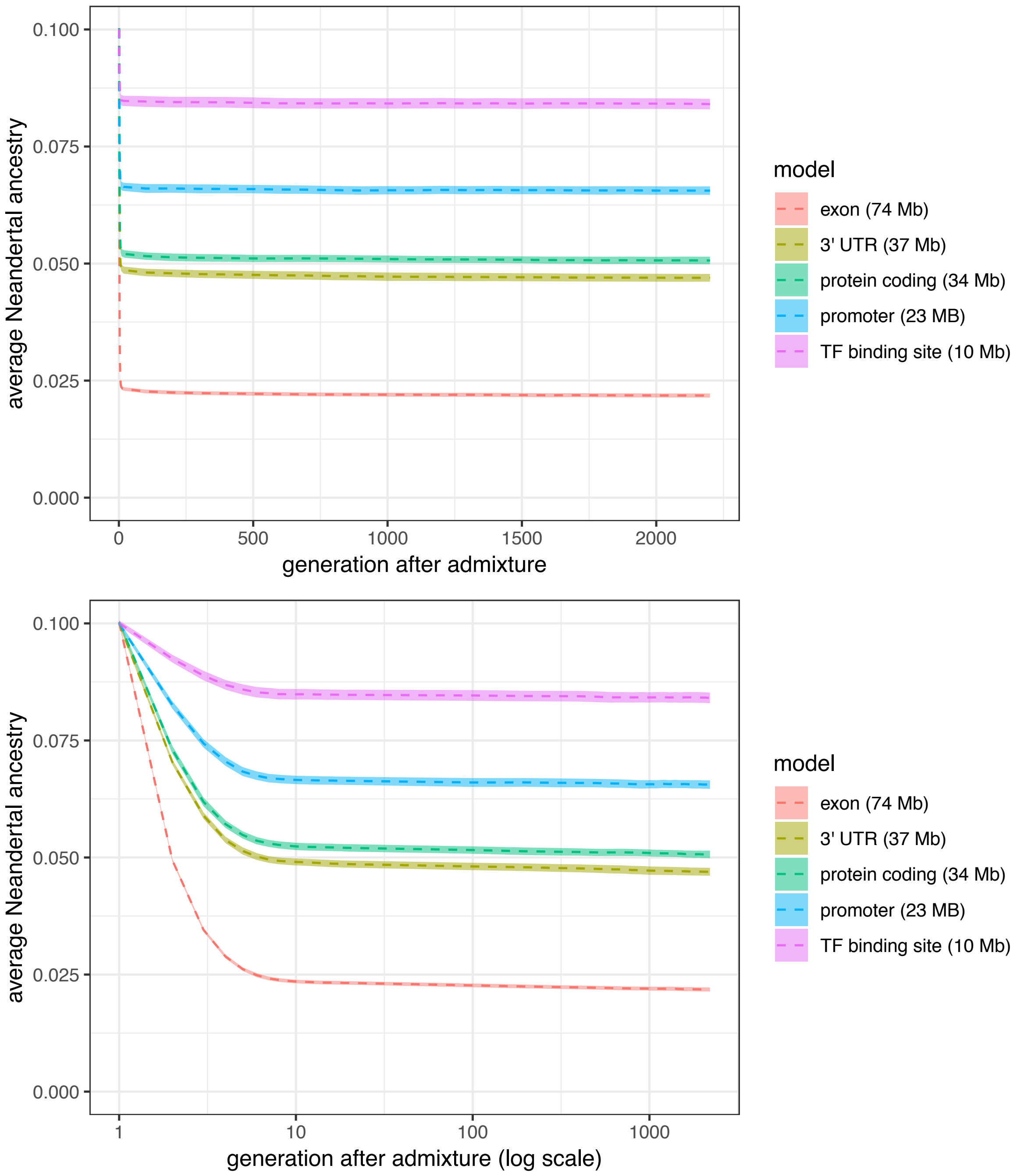

Fig. S12. The effect of changing the total amount of potentially deleterious sequence. Top and bottom panels show linear and logarithmic timescales, respectively. We simulated deleterious mutations in either full exonic, 3’ UTR, protein coding, promoter, or TF binding site regions. Simulations with larger “targets” for deleterious mutations have lower final levels of Neandertal ancestry. The shaded areas represent 95% confidence intervals.

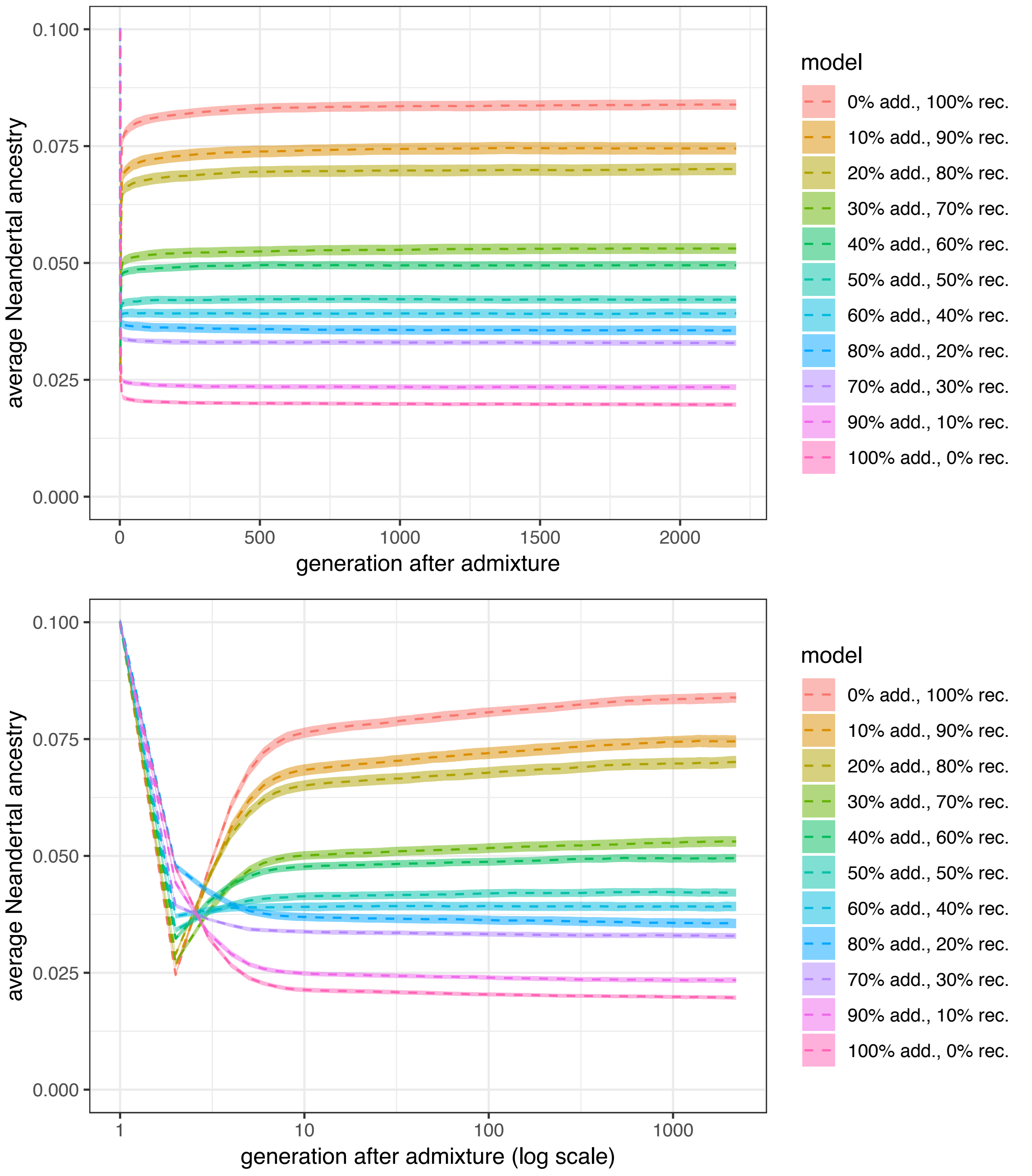

Fig. S13. The effect of changing the proportions of recessive and additive mutations. Top and bottom panels show linear and logarithmic timescales, respectively. It has been shown that the dominance coefficient of deleterious mutations can lead to Neandertal ancestry trajectories following entirely opposite patterns (4). Specifically, models with only recessive mutations lead to an initial increase of the Neandertal ancestry proportions due to heterosis (4). Due to these opposing effects of dominance, we investigated

scenarios with different mixtures of dominance coefficients of deleterious mutations. We found that changing the ratios of recessive and additive mutations affects only the final baseline of Neandertal ancestry in the population, and does not lead to a steady decline in Neandertal ancestry over time. The shaded areas represent 95% confidence intervals.

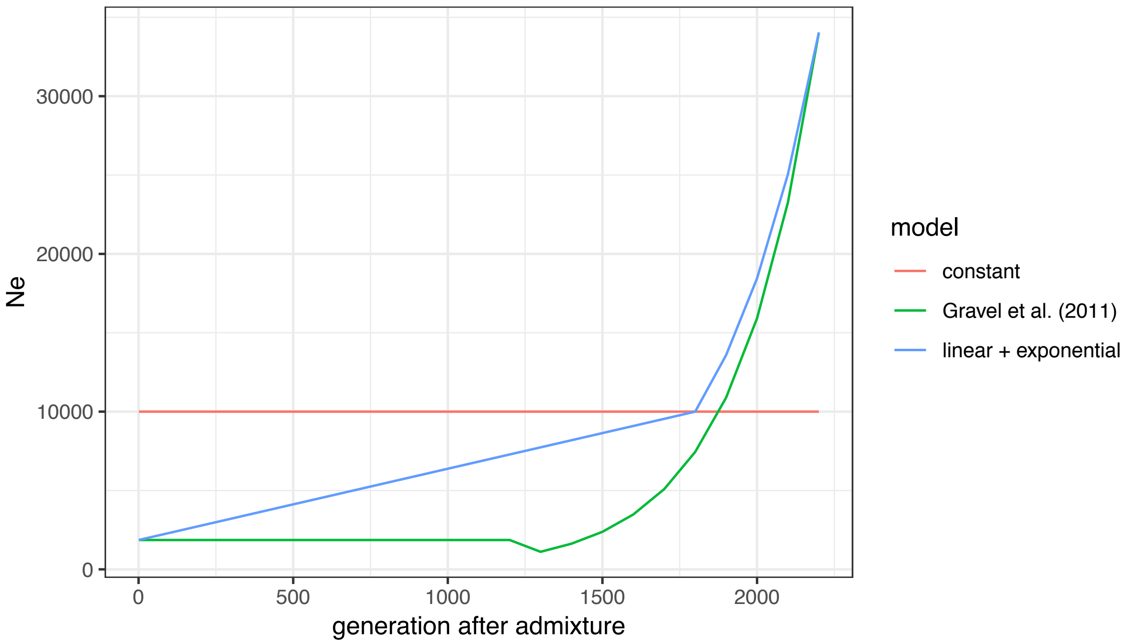

Fig. S14. Three models of non-African demography after Neandertal admixture. N_e as a function of time for three models of non-African demography: a model of constant N_e after Neandertal introgression, a model of initial slow linear growth post admixture, and a long bottleneck followed by a period of exponential growth (Gravel et al. (11)). Unless otherwise noted, all analyses in this paper used the constant N_e model.

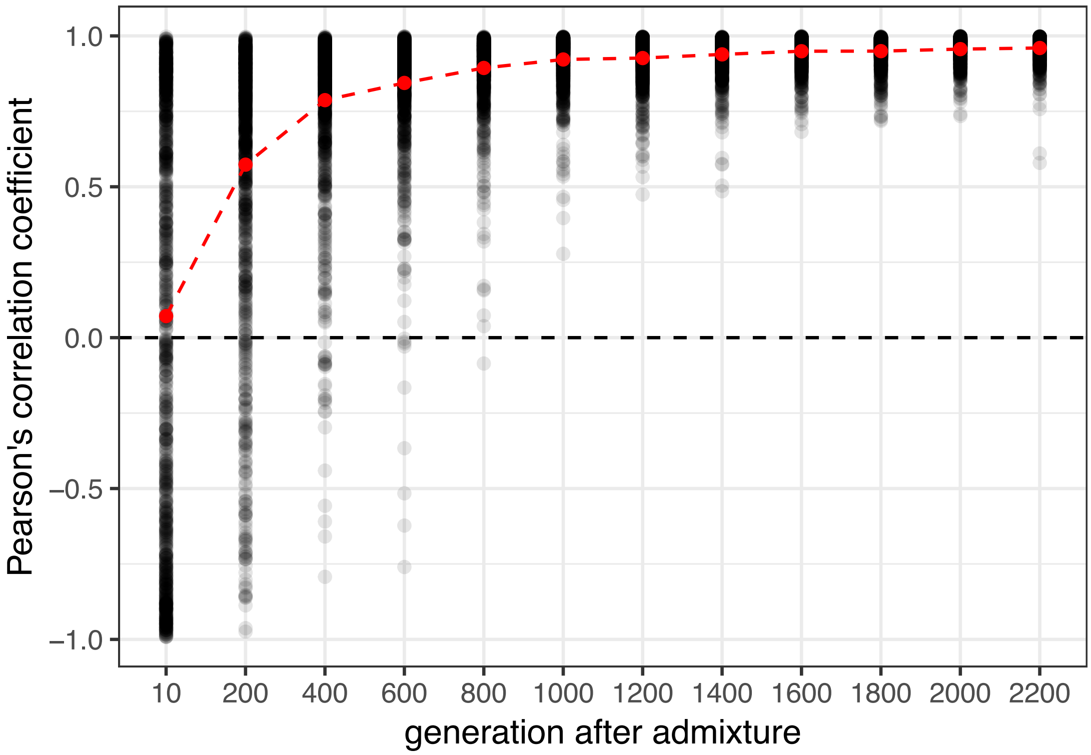

Fig. S15. Coefficients of correlation between the proportion of surviving Neandertal ancestry and distance to a genomic region under negative selection.
Pearson's correlation coefficient of a correlation between Neandertal ancestry proportion and the distance to the nearest region under negative selection, at a given point in time after introgression. Each black dot represents a correlation coefficient in a single simulated "individual" with 500,000 informative sites. Red dots indicate mean correlation coefficient at a given time-point. This figure uses the same data presented in Fig. 3C (which shows averages over all simulations at each individual time-point).

Table S1. Proportions of overlapping functional categories.

Each row contains proportions of overlap of a given region (row label) with all other annotated regions (column labels). CDS: protein coding sequence.

	protein coding	5' UTR	3' UTR	enhancer	promoter	phastCons
**protein coding**		0.050	0.174	0.003	0.051	0.615
**5' UTR**	0.163		0.039	0.005	0.402	0.244
**3' UTR**	0.157	0.011		0.007	0.009	0.256
**enhancer**	0.005	0.003	0.013		0.000	0.080
**promoter**	0.076	0.183	0.015	0.000		0.153
**phastCons**	0.206	0.025	0.095	0.000	0.034	

References

1. Kelleher J, Etheridge AM, McVean G (2016) Efficient Coalescent Simulation and Genealogical Analysis for Large Sample Sizes. *PLOS Computational Biology* 12(5):e1004842.

2. Fu Q, et al. (2015) An early modern human from Romania with a recent Neanderthal ancestor. *Nature* 524(7564):216–219.

3. Haller BC, Messer PW (2017) SLiM 2: Flexible, Interactive Forward Genetic Simulations. *Mol Biol Evol* 34(1):230–240.

4. Harris K, Nielsen R (2016) The Genetic Cost of Neanderthal Introgression. *Genetics*:genetics.116.186890.

5. Zerbino DR, et al. (2018) Ensembl 2018. *Nucleic Acids Res* 46(D1):D754–D761.

6. Siepel A, et al. (2005) Evolutionarily conserved elements in vertebrate, insect, worm, and yeast genomes. *Genome Res* 15(8):1034–1050.

7. Haeussler M, et al. The UCSC Genome Browser database: 2019 update. *Nucleic Acids Res*. doi:10.1093/nar/gky1095.

8. International HapMap Consortium, et al. (2007) A second generation human haplotype map of over 3.1 million SNPs. *Nature* 449(7164):851–861.

9. Eyre-Walker A, Woolfit M, Phelps T (2006) The Distribution of Fitness Effects of New Deleterious Amino Acid Mutations in Humans. *Genetics* 173(2):891–900.

10. Vernot B, Akey JM (2014) Resurrecting Surviving Neandertal Lineages from Modern Human Genomes. *Science* 343(6174):1017–1021.

11. Gravel S, et al. (2011) Demographic history and rare allele sharing among human populations. *PNAS* 108(29):11983–11988.

12. Lanfear R, Kokko H, Eyre-Walker A (2014) Population size and the rate of evolution. *Trends in Ecology & Evolution* 29(1):33–41.

13. Patterson N, et al. (2012) Ancient Admixture in Human History. *Genetics* 192(3):1065–1093.

14. Peter BM (2016) Admixture, Population Structure and F-Statistics. *Genetics*:genetics.115.183913.

15. Lazaridis I, et al. (2016) Genomic insights into the origin of farming in the ancient Near East. *Nature* 536(7617):419–424.

16. Lazaridis I, et al. (2014) Ancient human genomes suggest three ancestral populations for present-day Europeans. *Nature* 513(7518):409–413.

17. Mallick S, et al. (2016) The Simons Genome Diversity Project: 300 genomes from 142 diverse populations. *Nature* 538(7624):201–206.

18. Juric I, Aeschbacher S, Coop G (2016) The Strength of Selection against Neanderthal Introgression. *PLOS Genetics* 12(11):e1006340.
